# Strengthening Research and Practice in Community Health Systems: A Research Agenda and Manifesto

**DOI:** 10.34172/ijhpm.2021.71

**Published:** 2021-07-11

**Authors:** Moses Tetui, Anna-Karin Hurtig, Frida Jonsson, Eleanor Whyle, Joseph Zulu, Helen Schneider, Alison Hernandez, The Chaminuka Collective

**Affiliations:** ^1^School of Pharmacy, University of Waterloo, Waterloo, ON, Canada.; ^2^Department of Epidemiology and Global Health, Umeå University, Umeå, Sweden.; ^3^Department of Health Policy, Planning and Management, Makerere University School of Public Health, Kampala, Uganda.; ^4^School of Public Health & Family Medicine (SPHFM), University of Cape Town, Cape Town, South Africa.; ^5^School of Public Health, University of the Western Cape, Cape Town, South Africa.; ^6^South African Medical Research Council Health Services to Systems Unit, University of the Western Cape, Cape Town, South Africa.; ^7^Centre for the Study of Equity and Governance in Health Systems, Guatemala City, Guatemala.

**Keywords:** Community Health Systems, Priority Setting, Research Agenda, Multi-disciplinary, Diverse Contexts

## Abstract

While there have been increased calls for strengthening community health systems (CHSs), key priorities for this field have not been fully articulated. This paper seeks to fill this gap, presenting a collaboratively defined research agenda, accompanied by a ‘manifesto’ on strengthening research and practice in the CHS. The CHS research agenda domains were developed through a modified concept mapping process with a team of 33 experts on the CHS including policy-makers, implementers and researchers from institutions in six countries: Uganda, Guatemala, South Africa, Sweden, Tanzania and Zambia. The process began remotely with brainstorming research priorities and concluded in a one-week workshop that was held in Zambia where priorities for strengthening CHS were discussed, grouped into domains, interpreted, and drafted into a collective declaration. Eight domains of research priorities for CHSs were identified: clarifying the purpose and values of the CHS, ensure inclusivity; design, implementation and monitoring of strategies to strengthen the CHS; social, political and historical contexts of CHS; community health workers (CHWs); social accountability; the interface between the CHS and the broader health system; governance and stewardship; and finally, the ethical methodologies for researching the CHS. By harnessing a set of diverse and rich experiences and perspectives on CHS through a structured process, a multifaceted research agenda and manifesto that transcend context, disciplines and time were developed. We posit this as an entry into greater debate and diversity in the field as we continue to find ways to strengthen research and practice in the CHS.

## Introduction

Community health systems (CHSs) are the subject of growing interest based on their potential to leverage different community resources and advance population well-being. However, CHSs are understood in quite varied ways across the health systems’ research fraternity. These variations range from a restricted view of community health workforce endeavours or other local community volunteer programmes aimed at social accountability, to more generally understood concepts that encompass all of society’s efforts aimed at improving population well-being.^1^ As a relatively new and contested area of research, there is great potential to engage this growing interest and develop the field through initial research agenda setting.^2^ This is critical in generating dialogue and debate that can inform both inform both practice and further research.

Policy agendas such as the Millennium Development Goals and Sustainable Development Goals are implemented through research agendas among other ways. Research agendas are therefore essential in directing resources and coordinating efforts towards achieving set policy goals by creating logical frameworks through which channel scarce resources and actions can be used.^2,3^ Research agenda setting is approached in several ways including: the commissioning of small research activities; literature reviews; and convening of conferences, among others.^4^ Generally speaking, input from subject experts, engagement of a wide scope of stakeholders, building consensus and sharing of the research agenda to foster further debate are typical steps or processes in this endeavour.^5,6^ While some of these agenda-defining processes are aimed at to arriving at more inclusive research agendas, some are more focused on specific interests, often short-lived and limited in scope by disciplines and geographical reach. The latter often result in specific research questions that can easily be answered within a specific period of time and with resources that are usually readily available.^5^ For example, a recent literature and consultative review on global research agenda for CHSs largely focused only on community health worker (CHW) programmes.^3^ While this agenda is important, it does not adequately represent the scope of the field of CHSs, given that CHWs are just one of the components.^1^ Inclusive research agendas are more dynamic and create a stimulus upon which more long-term or enduring human interests are sustained is the kind of vision that our CHS research agenda setting is based on.

 In this paper, we aim to share a collaboratively defined agenda of CHS research priorities, crowned by a manifesto statement that emerged from the process. The participants in the agenda-defining process consisted of researchers from seven institutions, as well as front-line workers, managers and senior policy-makers from the Zambian Ministry of Health. Over the course of a one-week workshop, this collective leveraged their diversity (discipline, position and geography) to undertake a multidisciplinary exploration and mapping of research priorities for the CHS. This is envisioned to spiral into a more enduring and dynamic debate of shaping and reshaping CHSs that are responsive and accountable.

## Methods

In this section we present the process by which we developed the priorities that formed the basis for defining domains of the CHS agenda and the manifesto. A group of public health and health system players engaged in thinking, researching, policy-making and advocacy on CHSs met for a week (10–14 June 2019) at the Chaminuka Lodge in Lusaka, Zambia to collate our collective understandings of the CHS and generate a research agenda. The group of 33 participants included junior and senior health systems researchers from institutions in Zambia (8), South Africa (8), Sweden (4), Uganda (1), Tanzania (1), and Guatemala (1), as well as stakeholders from the Zambian Ministry of Health (4), community health workforce (3), and international partner organizations (3) (see Supplementary file 1).

**Figure 1:**
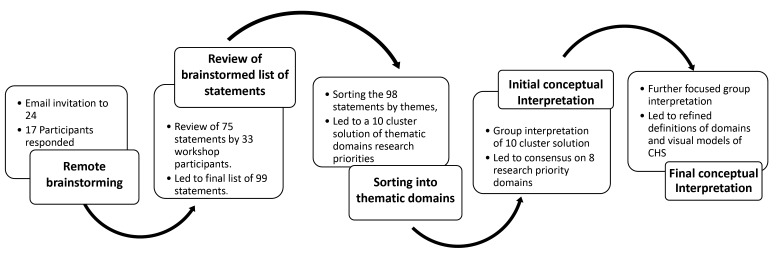
Modified Concept Mapping Process. Abbreviation: CHS, community health system.

A modified concept mapping process was used to engage participants in naming and integrating collective ideas about what is important for strengthening CHSs. Concept mapping is a structured, participatory method designed to visualize group thinking and facilitate consensus, and it is especially appropriate for involving multiple stakeholders in conceptualizing a complex topic in order to develop a theoretical framework, evaluation model, or agenda for policy or research.^7,8^ Our implementation of the method was guided by the steps outlined by Trochim and Kane with some adjustments in an effort to ensure that the process was inclusive and representative of the group’s collective ideas.^9^ Below are the steps we undertook (Figure 1).

###  Remote Brainstorming 

Prior to the workshop, a planning group including AKH, AH and FJ sent out email to the attendees, which asked them to return their ideas in response to the focus prompt statement –‘*In order to strengthen Community Health Systems, the research priorities I would like to see are….*’Responses were received from 17 participants and were compiled and consolidated, reaching a total of 75 unique statements after removing duplicates and combining statements with similar focus.

###  Review of Brainstormed List of Statements

When the workshop commenced, there were participants who had not had the opportunity to participate in the brainstorming by email. To ensure that everyone’s voice was included in the workshop’s collective efforts to conceptualize what is important to strengthen CHSs, all workshop participants took time to review the list of 75 statements of CHS research priorities and add statements if their ideas were missing. Based on notes from these small group discussions, some of the statements were edited for clarity and statements were added for a total of 98 idea statements (see Supplementary file 2).

###  Sorting Into Thematic Domains

 Participants were invited to sort the 98 ideas into groups based on themes that they perceived. This step was facilitated by the ‘groupwisdom’ software platform, where participants were asked to sort the statements into groups based on the theme of their focus and also name each thematic group. A total of 17 sorts were completed with participants working individually and in groups.

**Figure 2:**
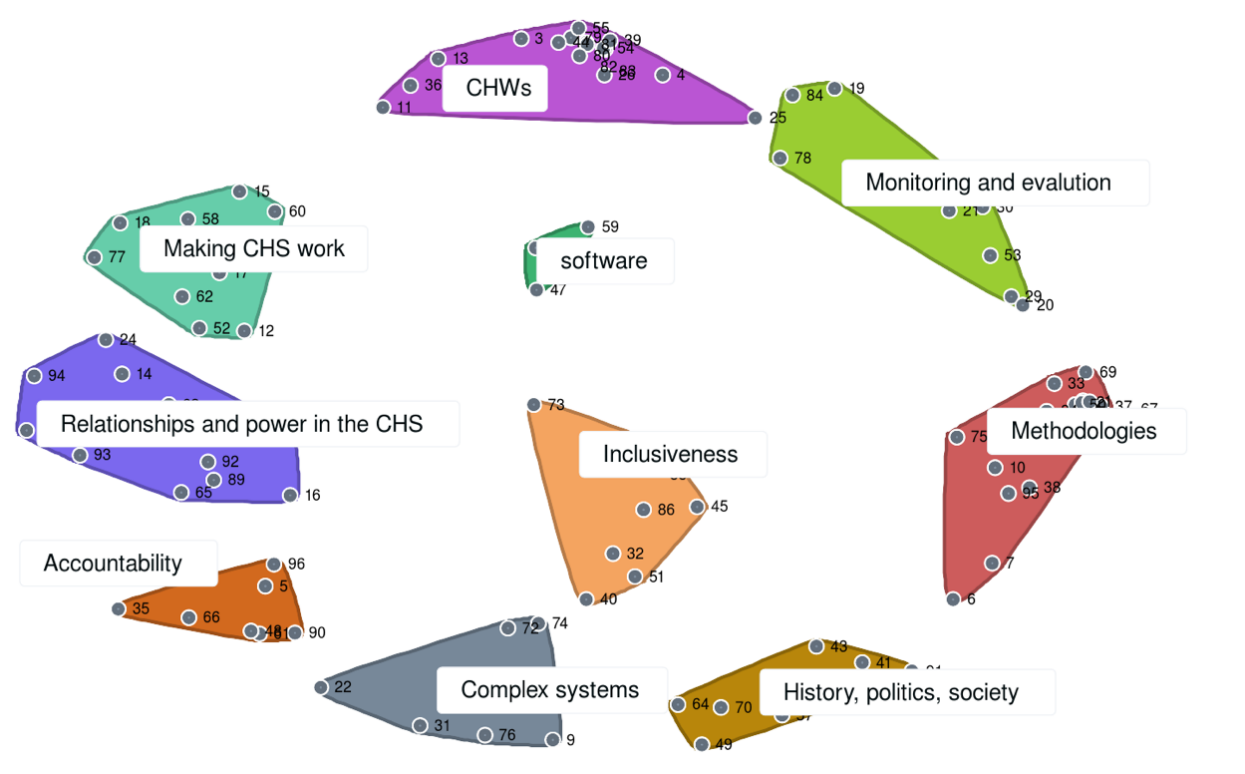
A “groupwisdom” Software Display of Initial Concept Map of 10-Cluster Solution of Thematic Domains of CHS Research Priorities Generated From Participant Sorting of the 98 Statements. Abbreviation: CHS, community health system.

Initial analysis of the sorting data enabled the team of concept mapping facilitators (IG, AH, FJ, EW) to gain an integrated view of how participants saw the statements fitting together in overarching themes. The idea statements were plotted on a point map (or distance matrix), where the statement points’ proximity to each other was based on the frequency with which they were grouped together. The point map was then partitioned into clusters of statements using hierarchical cluster analysis, and the team of facilitators examined how the points were grouped together into 6 to 15 clusters of ideas. At each level of clustering, the team assessed the coherency of the ideas that composed the clusters and whether the successive splitting of clusters added a valuable distinction for making sense of the themes represented in the ideas. They found that a 10-cluster solution was a good base for presenting the general domains and the ideas they contained to the broader group of participants (Figure 2).

###  Initial Concept Map Interpretation 

 On the third day, the participants were engaged in reviewing the concept map of the 10-cluster solution. Interpretation took place in small group discussions and a designated notetaker in each group recorded participants’ suggestions on how to make sense of the thematic domains that the clusters represented, and if they might be combined or divided to better capture important conceptual domains in CHSs research. The facilitator team reviewed the small groups’ interpretations of the clusters and found general consensus about the domains that were judged to be coherent and adequately capturing an important theme, and also significant overlap in the suggestions for modifying domains. Integration of the small-groups’ interpretations led to the identification of eight thematic domains organizing the statements.

###  Final Representation 

 On the fourth day, participants were engaged in further interpretation of the integrated list of thematic domains that emerged from the previous day’s discussions. In this step, the groups were charged with refining the definitions of the domains, developing a visual representation of how the domains fit together in a model or ‘landscape’ of CHSs, and a written declaration of how to strengthen CHS, drawing on their interpretation of the domains. Each of the four groups shared its visual model of the CHS domains and its declaration during a world cafe session later in the day. The lead workshop organizers later met with a small group of participants to synthesize the definitions of the priority research domains, reflect on the visual models and declarations coming out of the process, and draft an overall manifesto on research and practice for strengthening the CHS. The domains and the manifesto statement were then circulated to workshop participants for feedback.

## Results

The initial concept map in Figure 2 provided the jumping-off point for interpretive discussions that led to a general consensus on eight domains that captured the group’s collective ideas on priorities for strengthening CHS. These domains are defined and examples of the focus of statements within the domains are provided in Table. The first thematic domain, clarifying the purpose and values of CHS, emphasized the values of inclusiveness and equity and the approaches needed to promote them in the CHS. The second domain – design, implementation, monitoring and evaluation of strategies to strengthen the CHS – encompassed research priorities with a programmatic focus and evidence to inform decision-making. The domain of social, political and historical contexts underscored the need for critical thinking around the influence of intersecting contexts shaping the CHS and our approaches to it. The CHWs domain included focus on human resources issues, their performance, and their connection to communities. Social accountability, the fifth domain, covered priorities related to citizen participation, health committees, and responsiveness to the population. The domain called interface between the CHS and the broader health system encompassed practical issues for making the CHS work as well as the recognition of power dynamics in relationships at the interfaces. The governance and stewardship domain referred to priorities related to oversight and macro-level decision-making, while the eighth domain of ethical methodologies for researching CHS included priorities for conducting research that captures complexity and contributes to change.

**Table:** Priority Domains for a CHS Research Agenda, Their Definitions and Examples of Priority Topics Within Each Domain

**CHS Research Domain **	**Definition**	**Examples of Priority Topics **
Clarifying purpose and values of CHS	The core values, assumptions and principles that characterise our framing of the CHS and therefore of research on the CHS, such as equity, inclusiveness, whole of society approach, social determinants and locally driven.	Geared towards reducing inequalities Focused on intersectional needs Concern with the health of people (vs disease) Focused on the social determinants of health Takes into account perspectives of vulnerable groups
Design, implementation, monitoring and evaluation of strategies to strengthen the CHS	Decision-making and programmes to strengthen the CHS through all phases, from context-sensitive designs and models, the implementation and scale-up of programmes, and monitoring and evaluation strategies.	Different models of the CHS Examples of strong CHSs Strategies to guide programme implementation Scaling up locally driven innovations Monitoring at community level Performance indicators for the CHS Context sensitive evaluation strategies
Social, political and historical contexts	The history, political-economy, social and gendered contexts of the CHS at all levels, from global to local knowledge, beliefs and practices.	Historical development of CHS Politics and policy on the CHS Discourses on CHS Neoliberal reforms Gender relations CHS and the primary healthcare approach The CHS as nested in larger systems and society
CHWs	Holistic focus on the CHWs, including effective strategies for identifying, selecting and recruiting, training and developing, supporting and retaining CHWs.	Retention Motivation Training Roles on paper vs practice Embeddedness in communities Empowerment and agency Impact
Social accountability	Community accountability and responsiveness and participation mechanisms; strategies for collective action and effective citizen participation.	Participation mechanisms Responsiveness Collective action Power relations
Interface between CHS and the broader health system	Relationships and connections between CHSs and the broader health system.	Roles of boundary spanners Building trust between formal healthcare system and CHS Power dynamics within CHS Balance between formal sector and volunteers
Governance and stewardship	The oversight, direction and stewardship required to strengthen the CHS, ensure accountability, and promote inter-sectoral collaboration.	Resource allocation Involving private for-profit actors Intersectoral collaboration in CHS Overcoming fragmentation Partner coordination Community resource mobilization Sustainability
Ethical methodologies for researching the CHS	Methodologies and processes that align with the values of the CHS and contribute to social change.	Embeddedness Catching complexity Participatory action research Engaging with communities Contributing to change Co-producing knowledge Context sensitive

Abbreviations: CHS, community health system; CHWs, community health worker.

 Further conceptual interpretation of these domains in small groups was based on the same eight thematic domains, but participants employed different visual and metaphorical devices to depict their interrelationships. One model depicted them as elements of a self-sustaining ecosystem, another as systems within systems, while another arranged them as components of a building with a foundation of values and purpose and an overarching roof of governance. In another model, the domains were organized into micro-, meso- and macro-levels. Variations across the small-groups’ interpretations reflected varying mental models of the function of CHS and complex ideas surfaced about how domains of research priorities should fit together to strengthen them.

The CHS Chaminuka Manifesto, named after the venue of the workshop in Zambia (Box 1), represents the culmination of this multistep process of consultation and collective sense-making. As such, the manifesto draws from the interpretations of the thematic domains and the declarations on how to strengthen CHS written by the small groups to articulate a collective understanding of priorities and principles that should guide efforts to fortify CHSs, while accommodating multiple perspectives and starting points.

**Box 1. The Community Health System Chaminuka Manifesto**

We believe that communities are complex social systems with long histories, imbued with power relations that play out between people, families, neighbourhoods, committees and health workers. We understand CHSs as embedded in these community social systems and in broader health systems, with porous boundaries between them. The CHSs represent a significant health system asset or resource comprising both hardware elements, such as human resources, drugs and technologies, as well as software elements such as values, relationships and trust.

Health systems typically engage the CHS through CHW programmes. CHWs are mandated to provide primary and preventive health services, but both their mandate and their capacity to carry it out effectively are impacted by factors such as motivation, training, degree of embeddedness in communities they serve, and the disjuncture between their role as described on paper, and what is expected in practice. It is therefore vital that the research takes seriously the lived realities of CHWs, and seeks to establish effective strategies for identification, selection and recruitment of CHWs and best practice for training and continuous development of CHWs, enhance their agency, motivation and job satisfaction, and remove barriers to effective retention of CHWs in, and relationships with, the health system.

We believe that the CHS is also a site for the empowerment and participation of communities within broader health systems, allowing communities to hold government to account. This is to be achieved by strengthening community participation mechanisms that shift power relations, ensuring responsiveness of health systems to community needs, and actively pursuing strategies for collective action. This entails:

- Building capacity for collaborative governance and accountability, understood to include oversight, direction and stewardship to enable and strengthen the CHS, promote intersectoral collaboration, overcome fragmentation, ensure allocation of resources and build trust;
- Strengthening the interface and relationships between CHSs and their broader health systems, as well as between the community and health services;
- Recognizing the importance of addressing imbalances in power and building trust in relationships between stakeholders in strengthening the CHS.

We also believe that it is the responsibility of governments to support and strengthen the CHS, which includes:

- Working to overcome health system fragmentation
- Ensuring equitable resource allocation to CHSs
- Coordinating partnerships between actors within CHSs, and monitoring the impact of external partner actions on CHSs
- Ensuring balance of power between formal sector health workers and volunteer CHWs
- Recognizing the importance of boundary spanners, which mediate relations between CHSs and broader health systems
- Building trust between the formal health-care system and the CHS
- Strengthening the capacity of communities to hold government to account for maintaining their responsibility to the CHS.

We are explicit in positioning the values and principles underpinning our understanding and conceptualization of CHSs, and therefore our research in, on and for the CHS. Core to our values is the need to foreground marginal and vulnerable populations, and to fight to make those who are invisible, visible.

The values and principles we believe should be taken into account when designing, implementing, monitoring and evaluating the CHS are:

- A focus on reducing inequalities by acknowledging and striving to meet the intersectional needs of individuals as members of complex communities
- A concern with the health and well-being (including physical, mental and social well-being), rather than a disease-focused approach
- A commitment to locally driven solutions
- A whole-of-society perspective that seeks to harness the social determinants of health to promote well-being.

As such, we believe that research on, in, for and with CHSs should:

- Be inclusive
- Be locally driven and embedded in communities and societies
- Generate new knowledge through co-production
- Centre around community engagement through non-hierarchical participatory methodologies that foreground trust, balance of power, and strong and sustained interpersonal relationships
- Acknowledge the complexity and context-sensitive nature of CHSs, through a whole-of-society perspective
- Always be conducted with the intention of contributing to real-world social change
- Shift the centre of knowledge generation on the CHS to countries themselves, and in ongoing dialogue with policy-makers and practitioners.

In conducting this research, it is crucial to take account of, and research, the history, political-economy, and social and gendered contexts of the CHS, from global to local, including local knowledge beliefs and cultural practices. We understand this complex context not only as the context in which CHSs are embedded, but also as the context in which researchers conduct their work. In doing so, we recognize that global imbalances in knowledge generation may allow for certain ideas, interests and discourses on the CHS to dominate in ways that silence others.

The participants of the Chaminuka workshop resolve to continue building collaborations, research partnerships and community engagement platforms to strengthen CHSs. We further commit to conducting ethical, emancipatory research for and with all stakeholders towards inclusive empowered communities, while acknowledging the importance of histories, power and relationships, and using critical perspectives to understand the impact of these contextual factors on our daily work and relationships.

Abbreviations: CHS, community health system; CHWs, community health worker.

## Discussion

 This paper presents priority research domains and a manifesto declaration produced through a collaborative process to articulate an agenda for strengthening CHS. The process was enhanced by participants’ varied positionalities, which encouraged us to consider more carefully our starting points and assumptions on the CHS. For example, some of us were senior, others junior, others have focused on the role of CHWs in health systems, others on citizen mobilization and advocacy; some of us on health systems and their management, others on multisectoral development; some on macro-level policy and design, others on front-line action; some of us are researchers, other practitioners, and policy-makers. However, in terms of country level representation, the participants were largely from low- and middle-income countries and only country was represented from the high-income category of countries. While this posed a potential limitation to the priority and agenda setting process, the whole process was never made context specific, participants generally reflected on all their experiences in either context. A significant number of the participants had experiences with CHSs in high income countries through collaborations and lived experiences.

The process was facilitated by a modified concept mapping approach, which provided a structured and participatory tool for integrating our collective ideas about what is important for CHSs.^9^ In this application, the software-generated map of the thematic cluster solution served as a jumping-off point for further engaging participants in dialogue and interpretation, thereby raising contestation and generating an opportunity to find coherence and resolution through collective sense-making. By explicitly raising and harnessing different perspectives, we were able to stimulate rich thinking and a variety of representations of the CHS (textual, visual and metaphorical). Such an approach nonetheless is limited by the wealth of experience, depth and critical nature of the participants and dialogues undertaken.

 During the workshop, we challenged the idea of a single narrative on the CHS and emphasized the development of a multifaceted research agenda that could accommodate multiple perspectives and starting points cutting across generations, disciplines and context. We consider that the process was rich and robust enough to stimulate richer discussions around the development of the CHS field of research and practice, an iterative end that we welcome all to engage in as we build more responsive health systems around the world.

## Acknowledgments

 We acknowledge the funders of this study. In addition, we are grateful to all the workshop participants who freely shared their knowledge and CHSs experiences that enabled us to write this paper.

## Ethical issues

 All participants of the brainstorming and workshop sessions consented to participating the process and having their views shared through this publication.

## Competing interests

 Authors declare that they have no competing interests.

## Authors’ contributions

 MT led the drafting of the manuscript, AKH, HS, FJ, and AH supported MT in the drafting of the manuscript. In addition, AH provided overall technical oversight to the manuscript writing process. FJ, EW, and AH were a part of the lead team in the concept mapping process that was used to develop the content of the manuscript. JZ reviewed and provided technical guidance to the drafting of the manuscript. The Chaminuka collective actively participated in the priority setting process and their views are well captured in the manuscript. All authors reviewed and approved the manuscript for submission.

## Authors’ affiliations

^1^School of Pharmacy, University of Waterloo, Waterloo, ON, Canada. ^2^Department of Epidemiology and Global Health, Umeå University, Umeå, Sweden. ^3^Department of Health Policy, Planning and Management, Makerere University School of Public Health, Kampala, Uganda. ^4^School of Public Health & Family Medicine (SPHFM), University of Cape Town, Cape Town, South Africa. ^5^School of Public Health, University of the Western Cape, Cape Town, South Africa. ^6^South African Medical Research Council Health Services to Systems Unit, University of the Western Cape, Cape Town, South Africa. ^7^Centre for the Study of Equity and Governance in Health Systems, Guatemala City, Guatemala.

## Members of the Chaminuka Collective

 The Chaminuka Collective consists of the following members, displayed with their affiliations:

**Isabel Goicolea**; Department of Epidemiology and Global Health, Umeå University, Umeå, Sweden. **Charles Michelo**; School of Public Health, University of Zambia, Lusaka, Zambia. **Paul MalizganiChavula**; School of Public Health, University of Zambia, Lusaka, Zambia. **Adam Silumbwe**; School of Public Health, University of Zambia, Lusaka, Zambia. **KasapoChibwe**; School of Public Health, University of Zambia, Lusaka, Zambia. **Doreen Sitali**; School of Public Health, University of Zambia, Lusaka, Zambia. **Chama Mulumbwa**; School of Public Health, University of Zambia, Lusaka, Zambia. **MargarateMunakampe**; School of Public Health, University of Zambia, Lusaka, Zambia. **Wanga Zulu**; School of Public Health, University of Zambia, Lusaka, Zambia. **Miguel San Sebastian**; Department of Epidemiology and Global Health, Umeå University, Umeå, Sweden. **Uta Lehmann**; School of Public Health, University of the Western Cape, Cape Town, South Africa. **Tumelo Assegaai**; School of Public Health, University of the Western Cape, Cape Town, South Africa. **Leanne Brady**; School of Public Health & Family Medicine, University of Cape Town, Cape Town, South Africa. **Lance Louskieter**; School of Public Health & Family Medicine, University of Cape Town, Cape Town, South Africa. **Jill Olivier**; School of Public Health & Family Medicine, University of Cape Town, Cape Town, South Africa. **Marsha Orgill**; School of Public Health & Family Medicine, University of Cape Town, Cape Town, South Africa. **SiriliNathaneal**; School of Public Health and Social Sciences, Muhimbili University, Dar es Salaam, Tanzania.

## Supplementary files


Supplementary file 1. The Workshop Participants List.

Supplementary file 2. List of 98 Statements of Research Priorities Needed to Strengthen CHS.
