# Supplementary material for: Strengthening Research and Practice in Community Health Systems: A Research Agenda and Manifesto
Source: Int J Health Policy Manag. 2021 Jul 11;11(1):17–23. doi: 10.34172/ijhpm.2021.71 (PMC9278394; doi:10.34172/ijhpm.2021.71)
Supplement: Supplementary file 1 — The Workshop Participants List. [file ijhpm-11-17-s001.pdf]

Article title: Strengthening Research and Practice in Community Health Systems: A Research Agenda and Manifesto

Journal name: International Journal of Health Policy and Management (IJHPM)

Authors' information: Moses Tetui[1,2,3]*, Anna-Karin Hurtig[2], Frida Jonsson[2], Eleanor Whyle[4], Joseph Zulu[4], Helen Schneider[5,6], Alison Hernandez[7, 2], The Chaminuka Collective#

[1]School of Pharmacy, University of Waterloo, Waterloo, ON, Canada.

[2]Department of Epidemiology and Global Health, Umeå University, Umeå, Sweden.

[3]Department of Health Policy, Planning and Management, Makerere University School of Public Health, Kampala, Uganda.

[4]School of Public Health & Family Medicine (SPHFM), University of Cape Town, Cape Town, South Africa.

[5]School of Public Health, University of the Western Cape, Cape Town, South Africa.

[6]South African Medical Research Council Health Services to Systems Unit, University of the Western Cape, Cape Town, South Africa.

[7]Centre for the Study of Equity and Governance in Health Systems, Guatemala City, Guatemala.

#A full list of the investigators of the Chaminuka Collective is provided at the end of the article.

(*Corresponding author: mtetui@uwaterloo.ca)

Supplementary file 1. The Workshop Participants List

	Who	Role/Institution	Country
1	Charles Michelo	Dean, School of Public Health, University of Zambia (UNZA).	Zambia
2	Joseph Zulu	Assistant Dean, School of Public Health, UNZA.	Zambia
3	Doreen Sitali	Lecturer, School of Public Health, UNZA.	Zambia
4	Paul, Malizgani Chavula	Tutor, School of Public Health, Zambia.	Zambia
5	Chama Mulumbwa	PhD student, UNZA and Umeå University.	Zambia
6	Adam Silumbwe	PhD student, UNZA and Umeå University.	Zambia
7	Kasapo Chibwe	PhD student, School of Public Health UNZA.	Zambia
8	Margarete Munakampe	PhD student, School of Public Health, UNZA.	Zambia
9	Wanga Zulu	Chief Community Nursing Officer, Ministry of Health (MoH).	Zambia
10	Chila Simwanza	Assistant Director, Community Health Unit, MoH.	Zambia
11	Anderson Banda	Trainer, CHA Training School, MoH.	Zambia
12	Chansa Kafwimbi	CHA supervisor, MoH.	Zambia

13	Mildred Chisenga	CHA, MoH.	Zambia
14	Felix Chewe	CHA, MoH and President, CHA Association.	Zambia
15	Nampaka Nkumbula	Policy Manager, Innovations for Poverty Action – Zambia.	Zambia
16	Aldina Mesic	Senior Research Associate, Innovations for Poverty Action – Zambia.	Zambia
17	Olatubosun Akinola	Clinton Health Access Initiative.	Zambia
18	Dylan Edwards	Management Partner, MoH	Zambia
19	Anna-Karin Hurtig	HOD, Department of Epidemiology and Global Health, Umeå University.	Sweden
20	Isabel Goicolea	Associate Professor, Umeå University.	Sweden
21	Miguel San Sebastian	Professor, Umeå University.	Sweden
22	Frida Jonsson	Postdoctoral fellow, Umeå University.	Sweden
23	Alison Hernandez	Researcher, Centre for the Study of Equity and Governance in Health Systems.	Guatemala
24	Helen Schneider	Professor, School of Public Health, University of the Western Cape (UWC).	South Africa
25	Uta Lehmann	HOD, School of Public Health, UWC.	South Africa
26	Tumelo Assegaai	PhD student, School of Public Health, UWC.	South Africa
27	Eleanor Whyle	PhD student, School of Public Health & Family Medicine (SPHFM), UCT.	South Africa
28	Leanne Brady	Research and PhD student, SPHFM, UCT.	South Africa
29	Lance Louskieter	PhD student, SPHFM, UCT.	South Africa
30	Jill Olivier	Associate Professor, SPHFM, UCT.	South Africa
31	Marsha Orgill	Senior Lecturer and PhD student, SPHFM, UCT.	South Africa
32	Nathanael Sirili	Lecturer, School of Public Health and Social Sciences, Muhimbili University.	Tanzania
33	Moses Tetui	Senior Research Fellow, Department of Health Policy Planning and Management, Makerere University.	Uganda
