# Supplementary material for: Strengthening Research and Practice in Community Health Systems: A Research Agenda and Manifesto
Source: Int J Health Policy Manag. 2021 Jul 11;11(1):17–23. doi: 10.34172/ijhpm.2021.71 (PMC9278394; doi:10.34172/ijhpm.2021.71)
Supplement: Supplementary file 2 — List of 98 Statements of Research Priorities Needed to Strengthen CHS. [file ijhpm-11-17-s002.pdf]

Article title: Strengthening Research and Practice in Community Health Systems: A Research Agenda and Manifesto

Journal name: International Journal of Health Policy and Management (IJHPM)

Authors' information: Moses Tetui[1,2,3]*, Anna-Karin Hurtig[2], Frida Jonsson[2], Eleanor Whyle[4], Joseph Zulu[4], Helen Schneider[5,6], Alison Hernandez[7, 2], The Chaminuka Collective#

[1]School of Pharmacy, University of Waterloo, Waterloo, ON, Canada.

[2]Department of Epidemiology and Global Health, Umeå University, Umeå, Sweden.

[3]Department of Health Policy, Planning and Management, Makerere University School of Public Health, Kampala, Uganda.

[4]School of Public Health & Family Medicine (SPHFM), University of Cape Town, Cape Town, South Africa.

[5]School of Public Health, University of the Western Cape, Cape Town, South Africa.

[6]South African Medical Research Council Health Services to Systems Unit, University of the Western Cape, Cape Town, South Africa.

[7]Centre for the Study of Equity and Governance in Health Systems, Guatemala City, Guatemala.

#A full list of the investigators of the Chaminuka Collective is provided at the end of the article.

(*Corresponding author: mtetui@uwaterloo.ca)

Supplementary file 2. List of 98 Statements of Research Priorities Needed to Strengthen CHS

**No**	**Statements**
1	Methodologies for identifying community assets for local health systems.
2	Research methodologies to build trust.
3	Critical histories of CHS and CHW discourses.
4	Apply critical thinking to current practice.
5	Frameworks for considering marginality.
6	Theory-driven evaluation.
7	Cross-country case studies.
8	Embeddedness of researchers in communities and health systems strengthening.
9	Complexity-sensitive approaches that support context-embedded strategies.
10	Participatory action research.
11	Methodologies for identifying actors in the community health system.
12	Methodologies for identifying capabilities in the community health system.
13	Focusing on the experiences/stories of community health workers using qualitative methods.

14	Ones that recognize the value of co-producing knowledge with many stakeholders.
15	Generated with and for community members.
16	Intersectional approaches.
17	Visual methods.
18	Impact of visiting health workers on community health.
19	Surveillance of health problems and risks by community members.
20	Analysis of scaling up CHS interventions.
21	Strategies to guide implementation of CHS programs in different contexts.
22	Strategies to guide evaluation of CHS programs in different contexts.
23	Evaluation of community health worker interventions.
24	Performance indicators.
25	Monitoring in community health systems.
26	Strengthening outreach services through community health workers.
27	Cost-effective interventions to retain volunteering community health workers.
28	Perspectives of children.
29	Participation, what kind of participation is expected from communities in community health systems?
30	Focused on community health systems and not only community health workers.
31	Perspectives of disabled people.
32	Perspectives of women.
33	Perspectives of young people.
34	Focused on meeting people's intersectional needs.
35	Gender relations.
36	Adolescent sexual reproductive health in the community health system.
37	Focused on innovations.
38	The role of e-health in community health systems.
39	Software aspects of effective policy implementation.
40	Politics of and in community health systems.
41	Influence of local knowledge, beliefs, cultural practices on CHS strengthening.
42	Concerned with the health of people (vs those concerned with disease).
43	Geared toward reducing inequalities.
44	Questions of community health systems in urban contexts.
45	Resilience in the community health system.
46	Historical development of community health systems in different national contexts.
47	Discourses on community health systems.
48	Global conceptions of community health systems strengthening.
49	Different models of community health systems.
50	Community health systems and the primary health care approach.
51	Policies shifts on community health systems in the last decade.
52	Focused on community health system and the social determinants of health.

53	Ones that recognize that community health systems are nested in larger systems and society at large.
54	Examples of places with strong community health systems.
55	Cadres that are not chws.
56	Investigating the mismatch between what chws are trained and deployment and practice.
57	Health worker perceptions of community health systems programs.
58	Financial and indirect costs incurred by community health workers and other community actors.
59	Experiences of community health workers as members of task sharing strategies.
60	Embeddedness of community health workers in the communities they serve.
61	Formalization of community health workers.
62	Community health workers being involved in new tasks.
63	Training and supervision in community health systems.
64	Recruitment in community health systems.
65	The role of community health workers in strengthening retention of skilled health workers in rural areas.
66	The role of community health workers in reducing the double burden of diseases in low resource settings.
67	Defining the career paths of community health workers.
68	Incentives for motivating community health workers.
69	Community health workers satisfaction with their work.
70	Cost-effective interventions to retain community-based volunteers.
71	Health committees as accountability and participation mechanism.
72	Opportunities for locally driven innovation.
73	Social accountability.
74	Responsiveness of community health systems.
75	Strategies of collective action in the community health system.
76	How community participation can influence power relations.
77	Effective citizen participation in local and national health system accountability.
78	Overcoming fragmentation of initiatives (integration).
79	Intersectoral collaboration in community health systems.
80	Resource allocation to community health systems.
81	Partnership coordination and community health guidelines.
82	Community resource mobilization.
83	Sustainability of community health system.
84	Self-management in relation to community health structures.
85	The balance between the formal and volunteer health actors/sectors.
86	Building cooperation between the formal health care system and the range of players in the community health system.
87	Questions of integration of community and health systems.
88	Boundary spanners between formal and community health systems.

89	Power dynamics within community health systems.
90	Relationship building in the interface between health system management and community leadership.
91	How community health system that involves private for profit actors can work for all.
92	Building trust between the formal health care system and the range of players in the community health system.
93	Strategies of collaborative governance in the community health system.
94	How people have engaged policy and decision makers on the concept of community health systems.
95	Ways that policy makers can get better at listening and attending to the community.
96	Models of co-governance with shared ownership and power.
97	Power relations between community health workers and other stakeholders.
98	How community workers can engage and influence priorities and policy.
